# A common language: Cross-species network analysis reveals growth regulators

**DOI:** 10.1093/plphys/kiac417

**Published:** 2022-09-10

**Authors:** Rachel E Kerwin, Marieke Dubois

**Affiliations:** Department of Biochemistry and Molecular Biology, Michigan State University, East Lansing, Michigan 48824, USA; Department of Plant Biotechnology and Bioinformatics, Ghent University, Ghent, Belgium; VIB Center for Plant Systems Biology, Ghent, Belgium

Plants coordinate the growth of organs, such as leaves, through tightly regulated molecular and cellular processes, including cell proliferation and cell expansion (Kalve et al., 2014). Leaf growth is a dynamic process orchestrated by numerous genes called growth regulators, identified through their ability to alter growth rate and/or size when mutated or ectopically expressed. Growth regulators usually act at a particular stage, such as at the cell proliferation or cell expansion stage, and are expressed accordingly. For example, ANGUSTIFOLIA 3 (AN3), which stimulates cell proliferation, is highly expressed early during leaf development when cells are actively dividing but is expressed at low levels later when most cells are expanding or mature (Vercruyssen et al., 2014). Growth regulators that work together in the same (cellular) process are often co-regulated and can be identified based on shared expression patterns by analyzing transcriptomes. Such an approach has been used to identify growth regulatory gene networks in model species, including Arabidopsis (*Arabidopsis thaliana*) and maize (*Zea mays*; Baute et al., 2016; Vercruysse et al., 2020). However, when it comes to most crop species, our knowledge of plant organ growth is still incomplete.

Transferring genetic knowledge of complex processes like leaf growth and yield from lab-grown model species to field-grown crops often proves difficult. For example, in a recent large-scale study of hundreds of transgene constructs that increase yield and plant growth in lab-grown model species, only 4% could be confirmed in field-grown maize varieties (Simmons et al., 2021). While some core leaf growth-regulatory pathways are conserved between dicots and monocots—for example, those involving AN3, KLUH, or GROWTH-REGULATING FACTORS (Vercruysse et al., 2020)—identifying additional growth regulators that are translatable to crops is challenging, mainly because it is difficult to identify functional orthologs.

Orthologs are genes in different species that descended from a common ancestor. Having shared ancestry, orthologs tend to have conserved sequences, but may have diverged functions. Functional orthologs, then, are orthologs with similar functions in different species. The challenge of identifying functional orthologs is due, in part, to a complex gene duplication history that gave rise to a mottled gene copy number pattern across the plant kingdom, such that a gene from Arabidopsis, for example, may share two or more orthologs in a crop like maize. Discerning which orthologs are functionally conserved is further complicated by the fact that the pair with the highest sequence similarity may have diverged at the level of gene regulation. While a gene may be capable of a particular function, it can only do so if expressed in the right place at the right time. Thanks to an expansive (and expanding) collection of publicly-available transcriptomes, researchers now have the opportunity to incorporate cross-species spatiotemporal expression patterns into the search for functional orthologs, which should result in higher prediction accuracy.

In this issue of *Plant Physiology*, Curci et al. (2022) leveraged cross-species network analysis to identify functionally conserved, uncharacterized growth regulators. The authors obtained or generated leaf transcriptomes capturing active cell proliferation and cell expansion across Arabidopsis, maize, and aspen (*Populus tremula*). With this high (developmental) resolution dataset, the authors constructed co-expression networks for each species composed of sets of genes with similar expression patterns linked together into a large interconnected community. Individual networks, each generated using a different algorithm, were then aggregated to form robust metanetworks (Schiffthaler et al., 2021). Each species metanetwork was then split into five density subnetworks (DS), DS1, DS2, DS3, DS4, and DS5, by selecting the top 0.1, 0.5, 1, 5, and 10% most highly co-expressed genes, respectively. From the species metanetworks, the authors identified “triplets”: groups of orthologs, one from each species, with highly overlapping gene co-expression “neighborhoods” (Figure 1). The authors predicted that these triplets and their co-expressed gene neighbors have conserved functions.

**Figure 1:**
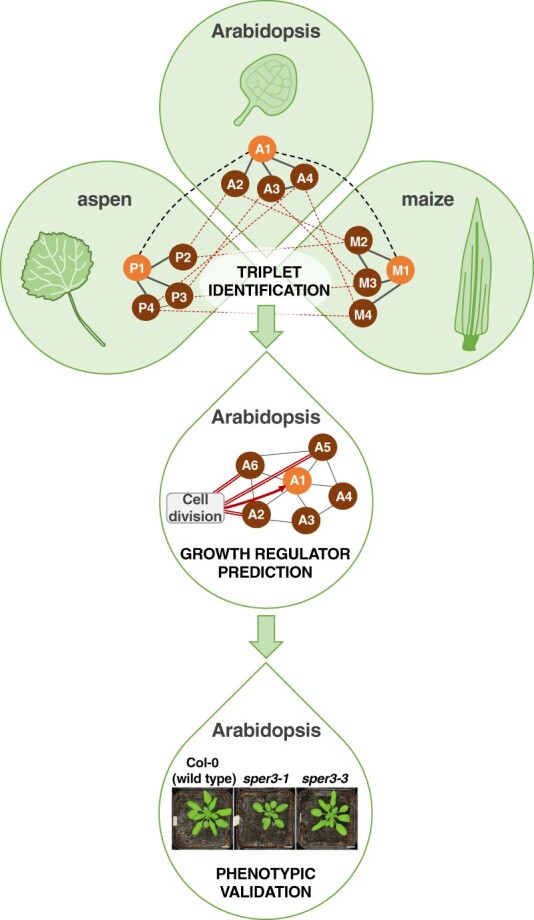
Cross-species network analysis identifies functionally conserved growth regulators. Leaf transcriptomes from Arabidopsis, maize, and aspen capturing active growth (i.e., cell proliferation and cell expansion) were used to generate co-expression networks for each species (grey solid lines in top panel represent co-expressed genes in each species). Cross-species expression conservation analysis revealed sets of triplets: orthologs, one from each species (thick black dashed lines in top panel), with highly overlapping gene co-expression “neighborhoods” (thin red dashed lines in top panel). In the depicted example, the genes A1 – P1 – M1 form a triplet (top panel). The authors identified the subset of triplets containing an experimentally-validated growth regulator from Arabidopsis, maize, or aspen. From this triplet subset, 82 Arabidopsis orthologs with peak expression during active growth were classified as growth regulators and used as guides to identify additional growth regulator candidates. Following a guilt-by-association approach, 2206 genes that were highly co-expressed with the guides were categorized as predicted growth regulators. In the depicted example, query gene A1 is co-expressed with A2, A3, A4, A5, and A6 (thin grey lines in middle panel). Among A1’s gene “neighbors”, A2, A5 and A6 are guides associated with a growth-related Gene Ontology term, cell division (red double lines in middle panel). Thus, A1 is classified as a predicted growth regulator (red arrow in middle panel). As proof-of-concept, the authors performed a detailed literature search that confirmed growth-related functions for 34 out of 61 (55.7%) of the top predicted growth regulators. Additionally, they performed mutant screening for nine of the top predicted growth regulators and observed leaf growth-related phenotypes for two of them (bottom panel). Together, these results validate the utility of cross-species network analysis for predicting bona fide growth regulators. Adapted from (Curci et al., 2022) Figure 1 and Figure 5C.

To prioritize triplets involved in leaf growth, Curci and colleagues filtered the triplets against a list of experimentally-validated growth regulators from Arabidopsis (71), maize (8), and aspen (71). Next, they identified the Arabidopsis orthologs from the triplet subset and filtered based on expression variation, retaining genes whose expression peaks during cell proliferation or cell expansion. This yielded a high confidence list of 82 “expression-supported” Arabidopsis leaf growth regulators. Functional enrichment analysis based on Gene Ontology classifications showed that the 82 growth regulators and their closest co-expressed gene “neighbors” in each density subnetwork were disproportionately assigned to processes such as cell division, cell cycle regulation, and cell expansion, consistent with their roles in growth regulation.

These 82 growth regulators were used as guides to mine additional candidates using a guilt-by-association approach (Figure 1). Across DS1 to DS5, the authors identified 2,206 genes that were highly co-expressed with the 82 guides and classified these genes as predicted growth regulators. To probe the reliability of these predictions, they compared their 2,206 predicted growth regulators against 391 Arabidopsis genes from the RARGE II database with experimentally-validated phenotypic effects on leaf traits when mutated (Akiyama et al., 2014). They found that the predicted growth regulators from DS1, the most stringent density subnetwork, recovered the highest proportion of validated RARGE II leaf trait genes. Next, for the top 100 genes from the list of 2,206 predicted growth regulators, the authors performed a manual literature search to look for evidence of their involvement in plant growth. Published studies were available for 61 of the top 100 predicted growth regulators. Notably, for 34 of the 61 (55.7%) published genes, leaf growth-related phenotypes were reported when knocked out or overexpressed, a hallmark of growth regulators. Lastly, the authors screened 9 of the 27 published mutants with no reported leaf phenotypes and observed altered rosette growth in two of them, further validating their computational growth regulatory predictions (Figure 1).

Overall, the study of Curci et al. (2022) demonstrates how cross-species expression analysis can enhance functional ortholog identification and uncover bona fide growth regulators. A similar strategy could be applied in crops for which transcriptome datasets are available to identify leaf growth regulators or regulators of other complex traits. Most agriculturally important traits, including seed yield or biomass, drought tolerance, nitrogen use efficiency, etc., are complex traits for which our knowledge on the genetics controlling them has many gaps. Considering our projected needs for increased food and feed, uncovering the genetic and expression networks behind these complex traits will be key to engineer plants that are more productive in a changing, and increasingly challenging, environment.

## Funding

R.E.K. is supported by funding from the National Science Foundation (IOS-1546617). M.D. is supported by a postdoctoral fellowship from the Flanders Research Foundation (FWO-12Q7919N).

*Conflict of interest statement:* None declared.
